# piRNA Profiling of Dengue Virus Type 2-Infected Asian Tiger Mosquito and Midgut Tissues

**DOI:** 10.3390/v10040213

**Published:** 2018-04-22

**Authors:** Yanhai Wang, Binbin Jin, Peiwen Liu, Jing Li, Xiaoguang Chen, Jinbao Gu

**Affiliations:** 1 Guangdong Provincial Key Laboratory of Tropical Disease Research, Department of Pathogen Biology, School of Public Health, Southern Medical University, Guangzhou 510515, Guangdong, China; wangyanhai79@126.com (Y.W.); jbb525@i.smu.edu.cn (B.J.); lpw111@i.smu.edu.cn (P.L.); lj123456@i.smu.edu.cn (J.L.); xgchen@smu.edu.cn (X.C.); 2 National Institute for Viral Disease Control and Prevention, Chinese Center for Disease Control and Prevention, Beijing 102206, China

**Keywords:** *Aedes albopictus*, piRNA, midgut, Dengue virus, antiviral immunity

## Abstract

The Asian tiger mosquito, *Aedes albopictus*, is a competent vector for the majority of arboviruses. The mosquito innate immune response is a primary determinant for arthropod-borne virus transmission, and the midgut is the first barrier to pathogen transmission. Mosquito antiviral immunity is primarily mediated by the small interfering RNA pathway. However, the roles that the P-element induced wimpy testis (PIWI)-interacting RNA (piRNA) pathway play in antiviral immunity in *Ae. albopictus* and its midgut still need further exploration. This study aimed to explore the profiles of both viral-derived and host-originated piRNAs in the whole body and midgut infected with Dengue virus 2 (DENV-2) in *Ae. albopictus*, and to elucidate gene expression profile differences of the PIWI protein family between adult females and their midguts. A deep sequencing-based method was used to identify and analyze small non-coding RNAs, especially the piRNA profiles in DENV-2-infected *Ae. albopictus* and its midgut. The top-ranked, differentially-expressed piRNAs were further validated using Stem-loop qRT-PCR. Bioinformatics analyses and reverse-transcription PCR (RT-PCR) methods were used to detect PIWI protein family members, and their expression profiles. DENV-2 derived piRNAs (vpiRNA, 24–30 nts) were observed in both infected *Ae. albopictus* and its midgut; however, only vpiRNA in the whole-body library had a weak preference for adenine at position 10 (10A) in the sense molecules as a feature of secondary piRNA. These vpiRNAs were not equally distributed, instead they were derived from a few specific regions of the genome, especially several hot spots, and displayed an obvious positive strand bias. We refer to the differentially expressed host piRNAs after DENV infection as virus-induced host endogenous piRNAs (vepiRNAs). However, we found that vepiRNAs were abundant in mosquito whole-body tissue, but deficient in the midgut. A total of eleven PIWI family genes were identified in *Ae. albopictus*; however, only AalPiwi5–7 and AalAgo3(1–2) were readily detected in the midgut. The characteristics of piRNAs in DENV-2-infected *Ae. albopictus* adult females were similar to those previously described for flavivirus infections but were not observed in the midgut. The reduced levels of vepiRNAs and incomplete expression of PIWI pathway genes in midgut samples from DENV-2-infected *Ae. albopictus* suggests that viral regulation of host piRNAs may not be an important factor in the midgut.

## 1. Introduction

Small RNA (sRNA) regulatory pathways (SRRPs) control essential aspects of antiviral immune responses to arboviruses in mosquitoes. There are three major SRRPs: canonical small interfering RNA (siRNA), endogenous microRNA (miRNA), and P-element induced wimpy testis (PIWI)-mediated transcriptional silencing pathways, which present in mosquitoes [1,2].

siRNAs, synthesized from double-stranded RNA (dsRNA) of natural endogenous or exogenous (e.g., viral) origin, are processed by cytoplasmic Dicer-2 (Dcr-2) and are loaded onto an Argonaute 2 (Ago2)-dependent RNA-induced silencing complex (RISC), leading to complementary mRNA degradation. In contrast to siRNAs, miRNAs are processed from long endogenous RNA transcripts, namely, primary miRNA transcripts (pri-miRNA), which are recognized and cleaved into precursor miRNAs (pre-miRNAs) in the nucleus through a microprocessor-complex composed of the ribonuclease III (RNase III) enzymes, Drosha and Pasha (partner of Drosha) [3]. Next, pre-miRNAs are transported into the cytoplasm, mediated by Exportin 5 (XPO5), accompanied by its cofactor, RanGTP, and then further cleaved by cytoplasmic Dicer-1 (Dcr-1, in fly, or Dicer in human) into mature miRNAs [4]. Mature miRNAs are loaded onto an effector Ago1-dependent RISC and guide the translational repression and the degradation of target transcripts [5]. The key roles of both the siRNA and miRNA pathways in mosquito antiviral immunity have been well characterized. On the other hand, PIWI-interacting RNA (piRNA)-mediated post-transcriptional regulation has been recently indicated to also involve an antiviral defense mechanism [6].

piRNAs (24–31 nucleotides in length) act as guardians of the genome, and cleave transposable elements (TEs or transposons) or messenger RNA (mRNA) that are involved in germ cell differentiation [7]. Further, recent studies have elucidated more detailed mechanisms regarding piRNA biogenesis. piRNAs mainly originate from long, single-stranded piRNA precursors (pre-piRNAs), which are generated in distinct genomic regions called piRNA clusters. In *Drosophila melanogaster*, the 5′ ends of pre-piRNAs were first trimmed using mitochondria-associated nuclease Zucchini (Zuc) [8,9,10], and then were loaded onto PIWI proteins and trimmed at their 3′ end by an unknown 3’–5′ exonuclease [11,12]. Finally, the 3′ termini of piRNAs was 2′-*O*-methylated using *Drosophila* methyltransferase DmHen1/Pimet to form mature PIWI-piRNA complexes or PIWI-piRISCs [11,12]. *D. melanogaster* expresses three PIWI proteins: P-element-induced wimpy testis (PIWI), Aubergine (Aub) and Argonaute 3 (Ago3) [13,14]. PIWI and Aub are mainly involved in primary piRNA biogenesis, and Aub- and PIWI-bound primary piRNAs show an antisense, and the 5′ ends show a uridine (1U) bias. Aub- and Ago3-piRNAs-induced silencing complexes (piRISCs) are then transported to the nucleus and cleave complementary target transcripts. This process generates a new sense piRNA with adenosine at position 10(10A), and sense-antisense piRNA pairs overlapping by precisely 10 nt at their 5′ ends. After being loaded onto Ago3 and 2′-*O*-methylated at the 3′ end, these secondary piRNAs go on to generate Aub-bound piRNAs using the same mechanism; this piRNA amplification loop is referred to as the “ping-pong” cycle [15].

The primary roles of piRNAs are to repress TEs in both the germ line and/or in somatic tissues, and this function is largely invariant across distant multicellular animal species [16]. piRNAs have recently also been associated with the innate antiviral response in insects. Indeed, for insects, virus infection-induced piRNAs were first observed in *Drosophila* ovarian somatic sheet (OSS) cells [17], and are also supposedly involved in host–pathogen interactions in virus-infected mosquitoes and mosquito cell lines [18,19,20,21,22,23,24,25,26,27,28,29].

On the basis of the difference in the origins of all of virus-induced piRNA, they fall into two groups: virus-derived piRNA (vpiRNAs) and virus infection induced host endogenous differentially expressed piRNAs (vepiRNAs) [22]. Virus-derived piRNAs are grouped into virus-derived small RNAs (vsRNAs), which originate from both viral RNA strands with an inhomogeneous distribution across the complete viral genome. vsRNAs mainly contain virus-derived small interfering RNAs (vsiRNAs) and virus-encoded miRNA (vmiRNA). virus-derived small interfering RNAs from viral-folded structures are processed by Dicer-like proteins (DCLs) or double-stranded RNA (dsRNA), which are involved in the RISC assembly pathway, and lead to sequence-specific degradation of target single-stranded viral RNA [30]. Virus-encoded miRNAs are derived from highly-structured, single-stranded regions of transcripts, originating predominantly from DNA viruses, which are supposed to regulate the expression of host and/or specific viral mRNAs [31,32]. The origin of vpiRNA is not completely clear; however, in some viruses, this pathway could also be involved in the ping-pong mechanism, such as with the host cell native piRNA [6]. Furthermore, Li et al. were the first to characterize Ultrashort RNA (usRNA, also unusually-small RNA, 13–19 nt in length) in Kaposi’s sarcoma-associated herpesvirus (KSHV)-infected cells [33]. These usRNAs were also characterized in dengue virus (DENV)-infected *Aedes* mosquitoes [20,25], as well as in bunyavirus La Crosse virus (LACV)- and Sindbis virus (SINV)-infected mosquito cells [19]. However, the role of usRNAs in the antiviral immune response still needs to be further elucidated.

*Aedes albopictus* (Asian tiger mosquito), considered one of world’s worst invasive alien species, which also has an aggressive biting behavior, has great potential to transmit multiple virus infections. As a secondary vector of dengue viruses, *Ae. albopictus*-borne dengue fever (DF) and dengue hemorrhagic fever (DHF) are still major threats to human health in tropical urban areas of Asia [34,35]. Transmission of DENV through *Aedes* requires a virus to effectively invade and replicate in midgut epithelial cells, disseminate into the hemocoel from the midgut, and move to the salivary gland acini [36]. Thus, the midgut is the first barrier to pathogen transmission. Furthermore, SRRPs have also been found in the midguts of West Nile virus (WNV) orally-exposed *Culex pipiens* [37]. Therefore, further investigation is required to determine whether the piRNA pathways are broadly involved in the antiviral response in *Ae. Albopictus*; their role in the midgut also needs to be investigated.

In this study, we used a deep sequencing approach to characterize that *Ae. albopictus*, infected with dengue virus type-2 (DENV-2), produces both vpiRNAs and vepiRNAs. We then determined whether specific piRNAs are produced in mosquito whole body and midguts in response to infection. This will significantly enhance our understanding of the relationship between the piRNA pathway and the mosquito innate immune response to virus infections.

## 2. Materials and Methods

### 2.1. Mosquitoes

The experiments were performed using the *Ae. albopictus* Foshan (Guangdong, China) strain, which was kindly provided by the Center for Disease Control of Guangdong (CDC, Guangdong), China, and established in the laboratory in 1981. All mosquitoes were reared in a bioclimatic insectary at 25 ± 1 °C, with 70–80% relative humidity, under a 10:14 h daily light–dark (LD) cycle. Larvae were fed with a mixture of dry yeast powder and fish food (Yee^®^, Shandong, China) at a ratio of 1:3, and adults were fed with a sugar solution (10% glucose). Adult female mosquitoes were allowed to take blood by feeding on an anesthetized mouse, which was then returned to its cage.

### 2.2. Virus Strains and Infection

The DENV-2 New Guinea C strain (NGC) was used in this study and was obtained from Sun Yat-sen University Zhongshan School of Medicine. DENV-2 stocks were obtained by inoculating a nearly confluent monolayer of C6/36 cells in a 25-cm^2^ tissue culture flask, with the virus diluted to 1:5 in 1 mL RPMI-1640 medium (Gibco; Thermo Fisher Scientific, Inc., Waltham, CA, USA) containing 2% heat-inactivated fetal bovine serum (FBS; Gibco; Thermo Fisher Scientific, Inc.). After five days in culture, the cells and supernatant were subsequently harvested via gentle pipetting. Then, the cells were pelletized using centrifugation at 3000 rpm for 15 min, and the viral supernatant was harvested and adjusted to 20% FBS, aliquoted, and then stored at −80 °C. The viral titers were titrated using a viral plaque formation on BHK-21 cells. Results showed that the virus titer was 10^6.85^ plaque-forming units per ml (PFU)/mL.

*Ae. albopictus* adult females (4–6 days old) were starved for 24 h and then moved to 2.5 L disposable paper feeding containers. Adult females were fed with defibrinated sheep’s blood (Applied Biological Products Management, Aldinga Beach, Australia) mixed with DENV-2 viral supernatant (5:1). Prior to feeding, blood–virus mixtures were incubated at 37 °C for 1 h using glass membrane feeders covered by a porcine intestinal membrane [38]. After feeding, mosquitoes were briefly anesthetized using CO_2_; non-engorged and partially-fed mosquitoes were discarded. The control mosquitoes were exposed to uninfected bloodmeal.

### 2.3. Mosquito Tissue Dissection and RNA Isolation

DENV-2-positive mosquitoes were identified by reverse-transcription PCR (RT-PCR) with DENV-2-specific primers at 9 days post-infection (dpi). Mosquito forelegs were removed, and RNA was extracted using TRIzol Reagent (Invitrogen; Thermo Fisher Scientific, Inc.). RT-PCR was employed to identify infected mosquitos with DENV-2-specific primers [39] (Table S1). The RT process was performed using the SuperScript^®^ III first-strand synthesis system (Thermo Fisher Scientific, Invitrogen, Carlsbad, CA, USA), and only DENV-2-positive mosquitoes were used for subsequent sRNA library construction.

Pools of three groups of infected/uninfected midguts (*n* = 100 per group) were collected at 1 dpi by dissecting anesthetized DENV-2-positive mosquitoes onto a drop of pre-chilled physiological saline, and the slide was then placed on a dissecting microscope. Midguts were placed into RNase-free 1.5 mL microcentrifuge tubes containing 1 mL of RNAlater (Life Technologies, Thermo Fisher Scientific, Walthman, MA, USA) and were then flash frozen in a dry ice/ethanol bath and stored at −80 °C until subsequent RNA extraction. Total RNA from each group was extracted with Trizol^®^ Reagent, according to the manufacturer’s protocol. For the DENV-2-infected/uninfected adult female samples, pools of three groups (*n* = 50 per group) of whole mosquitoes were combined, frozen in liquid nitrogen, and then ground to a fine powder for RNA extraction using TRIzol^®^ reagent.

### 2.4. sRNA Library Construction and Sequences Analysis

The primary objective of this study was to profile the small RNA in DENV-2 infected mosquitos and midguts. Instead of constructing individual libraries for each replicate, 6 ng of total RNA per biological replicate in each sample (3 replicates) was pooled together for subsequent sRNA library construction. The small RNAs were purified using 15% denaturing polyacrylamide gel electrophoresis to enrich the desired size range (13–30 nt), and were sequentially ligated with 5′-(5GUUCAGAGUUCUACAGUCCGACGAUC) and 3′- (5PUCGUAUGCCGUCUUCUGCUUGUidT) end RNA oligonucleotide adaptors; then, they were subsequently reversely transcribed with the small RNA RT-Prime (5′-CAAGCAGAAGACGGCATACGA-3′), and the adapter sequence specific primers were used to amplify the inserted DNA using PCR. After cluster generation of purified PCR products (forward primer: 5′-AATGATACGGCGACCACCGACAGGTTCAGAGTTCTACAGTCCGA-3′; reverse primer: 5′-CAAGCAGAAGACGGCATACGA-3′), library preparations were sequenced using an Illumina Genome Analyzer (Illumina, San Diego, CA, USA). All sequencing was carried out at the Beijing Genomics Institute (BGI) (Shenzhen, China). Raw sequence reads were submitted to the NCBI Short Read Archive (SRA) under the accession numbers SRA129084 and SRP049501.

### 2.5. Sequence Analysis

The adaptor sequences were removed, low-quality tags were filtered out, and the contamination of adaptor–adaptor ligation was cleaned up with Trimmomatic-0.30 using default parameters [40]. Unique reads of 24–30 nt were selected for further analysis and were mapped to the genome assembly of the *Ae. albopictus* Foshan strain (version AaloF1), as provided in VectorBase [41], using the Short Oligonucleotide Analysis Package 2 (SOAP2) [42]. Furthermore, reads matching noncoding tRNAs, snoRNAs, rRNAs, small nuclear RNAs (snRNAs), smiRNAs, and exons of protein-coding genes were removed; additionally, reads containing poly-A/T/C/G nucleotides (a minimum of eight homopolymer repeat nucleotides) were excluded. The remainder of the genome-matched reads were used as piRNA-like sRNA candidates for subsequent analyses.

### 2.6. Ping-Pong Signature

Ping-pong signals were defined as a typical pair of sense and antisense piRNAs, overlapping by 10 nt at their 5′ ends [43]. To confirm the fraction of piRNAs involved in ping-pong pairs, we first counted all the uniquely-matched piRNAs; each piRNA was then matched to the database of piRNAs in the pool. Complimentary piRNAS matching along the 5′ ends were considered a pair. We counted the length of paired bases (the last paired nucleotide position relative to the 5′ ends of the piRNAs) of every partner. Finally, the paired piRNAs were classified according to the length of overlapped paired bases, as previously described, and a 10 bp overlap was defined as a typical ping-pong signature [44].

### 2.7. PIWI Protein Family Members’ Ortholog Identification

*Ae. albopictus* PIWI family genes were identified by a TBLASTX search of the *Ae. albopictus* transcripts and genome [41] using the *Ae. aegyti* PIWI family genes (Armitage: AAEL010693, AAEL010696; Spindle-E: AAEL013235; Rm62-like: AAEL001317, AAEL001769, AAEL002083, AAEL002351, AAEL004978, AAEL008738, AAEL010402, AAEL010787-PA, AAEL013985; Ago3: AAEL007823; Ago4-like: PIWI1 AAEL008076, PIWI2 AAEL008098, PIWI3 AAEL013692, PIWI4 AAEL007698; Ago5-like: PIWI5 AAEL013233, PIWI6 AAEL013227, PIWI7 AAEL006287) as search queries [45].

### 2.8. Stem-Loop qRT-PCR Analyses

Candidate piRNAs were reverse transcribed to cDNA per biological replicate using specific stem-loop RT primers (Table S2). Quantitation was performed on an ABI 7500 Fast Real-Time PCR system (Applied Biosystems Life Technologies, Foster City, CA, USA) using cDNA specific forward primer and a universal reverse primer, as listed in Table S2. U6 small nuclear RNA (U6 snRNA) was used for normalization in all samples. The specificity of amplification was confirmed by a melting curve analysis and also by running PCR products on agarose gels (3%). The quantitative RT-PCR analysis was performed three times using independent purified RNA samples with three replicates for each sample.

### 2.9. Phylogenetic Analyses

Putative amino acid sequences of full-length PIWI subfamily proteins were aligned using Clustal X software [46] (version 1.81) using the default parameters. Phylogenetic relationships among proteins were estimated using the neighbor-joining (NJ) method using the MEGA 4 package [47]. The reliability of the resulting topologies was tested using the bootstrap method [48] with 1000 pseudoreplicates.

### 2.10. Gene Expression Analyses of the PIWI Protein Family

To explore the expression profiles of the PIWI protein family gene in *Ae. albopictus*, time series transcriptome data from the midguts of DENV-2-infected adult females and carcasses of *Ae. albopictus* (three replicates per sample, *n* = 50–70 per replicate)(GenBank SRA database accession number SRP077936) were used to analyze the gene expression levels [49]. Clean reads were aligned to the *Ae. albopictus* Foshan strain genome reference with Bowtie2 (version 2.3.4.1) [50] and then the sequencing reads for each RefSeq gene were calculated using RSEM (version 1.273) [51] to estimate the expression levels. The trimmed mean of the M-values normalization method (TMM) was used to normalize the gene expression levels in different libraries [52]. Finally, the transcript expression levels were compared, based on transcripts per million transcripts (TPM). The TPM can be seen as counts and therefore, as Poisson distributed values. A test for differences in TPM was found in two experimental conditions based on Poisson distribution statistics [53].

The gene expression profiles were further confirmed using RT-PCR. Total RNA was extracted from *Ae. albopictus* adults from different tissues (whole body and midgut), and at different developmental stages (0–24 h embryos, 24–48 h embryos, 1st–2nd larvae, 3rd–4th larvae, pupae, 2 days post-emergence sugar meal adult males and females, 24 h and 48 h post blood feed adult females) using TRIzol Reagent (Invitrogen, Carlsbad, CA, USA). Any residual DNA was removed using a TURBO DNA-free™ Kit (Ambion, Life Technologies, TX, USA). First-strand cDNA was synthesized from the total RNA using Oligo (dT) primers and a RevertAid First Strand cDNA Synthesis Kit (Thermo scientific). PIWI protein family genes were detected by RT-PCR using sequence-specific primers. *Ae. albopictus* ribosomal protein 7 gene (*AalrpS7*) (GenBank: JN132168) was used as a control. The sequences of the primers used in the RT-PCR are shown in Table S1. The amplified products of RT-PCR were separated on 1.0% or 2.0% agarose gels, depending on the size of the product, and purified using an E.Z.N.A Gel Extraction Kit (Omega Bio-tek, Doraville, GA, USA). These cDNA fragments were cloned into a plasmid pGEM-T Easy Vector (Promega, Madison, WI, USA) and confirmed via sequencing (Beijing Genomics Institute, BGI, Beijing, China).

## 3. Results

### 3.1. sRNA Seq Analyses

**Figure 1:**
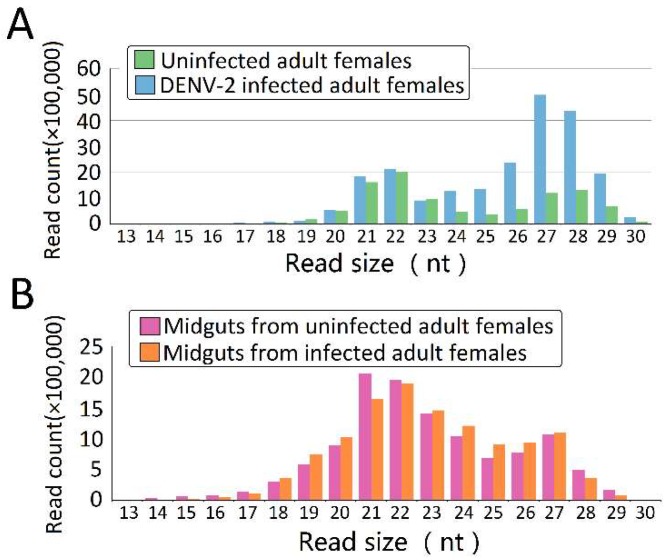
The nucleotide length distribution of sequence tags obtained from the four *Aedes albopictus* small RNA (sRNA) libraries. Size distribution and relative frequency in each sample are shown for the small RNAs derived from (**A**) uninfected adult females and dengue virus type-2 (DENV-2)-infected adult females; and (**B**) midguts from uninfected and DENV-2-infected adult females. Abbreviations: nt, length of small RNA read in nucleotides.

In order to explore the DENV-2-induced expression profiles of piRNAs in *Ae. albopictus* and midguts, we sequenced four libraries from uninfected adult females, DENV-2-infected whole adult females, and midguts from uninfected and DENV-2-infected adult females. After trimming the adaptors, cleaning up contaminations, and excluding low-quality reads, clear reads were obtained from four different libraries (Table S3). Both uninfected female adults (Figure 1A) and uninfected female midguts (Figure 1B) showed a major peak at 22 nucleotides, accompanied by a sub-peak at 26–28 nucleotides; in DENV-2-infected female adults, the 22 nt class was the secondary peak, and the primary peak shifted to 27 nt, which may represent PIWI-interacting RNAs (piRNAs) (Figure 1A).

### 3.2. Molecular Characterization of DENV-2-Derived sRNAs

DENV-2-derived small RNAs (DENV-2 vsRNAs) were identified by aligning the small RNA library of DENV-2-infected adult females and DENV-2-infected female midguts to the DENV-2 genome through pattern matching using SOAP2 (Table S4). The sRNA segments (13–30 nt), of which sequences showed complete matches to the DENV-2 genome at each base position, were considered to be vsRNAs. vsRNAs are distinguishable from host-derived sRNAs in that they may show imperfect complementarity to sites in the minus- or plus-strand of the viral genome. The analysis results show that the DENV-2-infected adult female libraries had 11,716 reads, representing 6345 unique perfectly-matched DENV-2 vsRNAs, accounting for only 5.60 × 10^−2^ percent of the total sRNA reads. The DENV-2-infected female midgut libraries displayed the number of vsRNAs (44,494 reads and 14,885 unique reads), and accounted for 37.41 × 10^−2^ percent of the total abundance of sRNA reads, possibly due to the rapidly-increasing levels of viral proliferation in the midgut, or to the insulation of the dsRNA trigger in cytoplasmic membrane-enclosed vesicles [54,55,56]. A small number of vsRNAs perfectly mapped both the viral and host genomes (Table S4), which matched the *Ae. albopictus* genome in a random manner, due to the complete nature of sRNA homology for both viral and host genomes; thus, it is difficult to discover the origin of this micro group of sRNAs.

**Figure 2:**
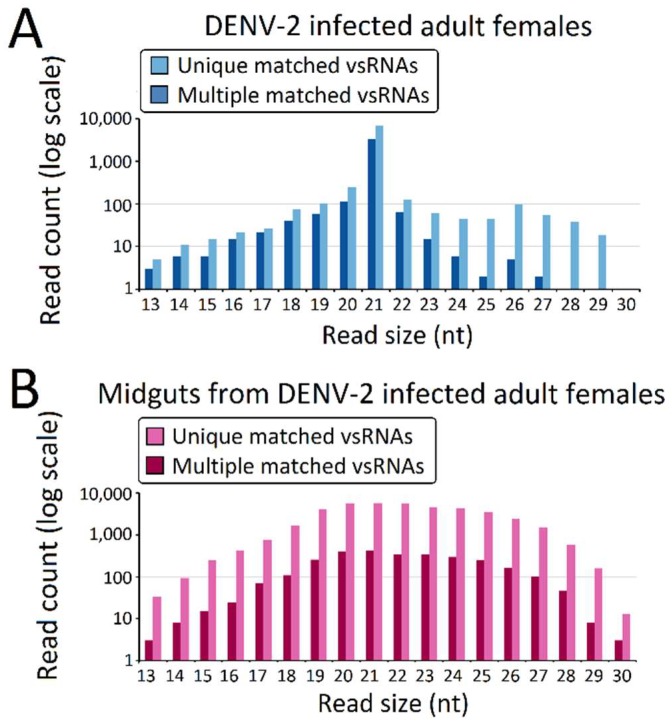
Virus-derived small RNAs (vsRNAs) size distribution varies among dengue virus 2 (DENV-2)-infected adult females and midguts from DENV-2-infected adult females. Size distribution and relative frequency in each sample are shown for the DENV-2-specific small RNAs (vsRNA) derived from (**A**) DENV-2-infected adult females and (**B**) midguts from DENV-2-infected adult females. Abbreviations: nt, length of small RNA read in nucleotides. Note the logarithmic scale on the Y-axes.

The predominant vsRNA sizes, both in DENV-2-infected adult females (Figure 2A) and DENV-2-infected female midgets (Figure 2B), were grouped into 21 nt (vsiRNA), and were considered to be the size characterization for DCL products. However, in contrast to the 21 nt vsRNA percentage (89.3% of vsRNA) in the DENV-2-infected adult female libraries, 21 nt vsRNA only occupied 14.61% of vsRNAs reads in the DENV-2-infected female midgut libraries. Though vsRNAs in DENV-2-infected adult females also displayed a sub-peak at 26 nt, these 24–30 nt length vsRNAs were less than 3% of the abundance of DENV-2-infected adult female vsRNAs. DENV-2-infected female midgut vsRNAs displayed an approximate Gaussian distribution, and the 24–30 nt length sRNAs corresponded to 31.25% of the reads.

**Figure 3:**
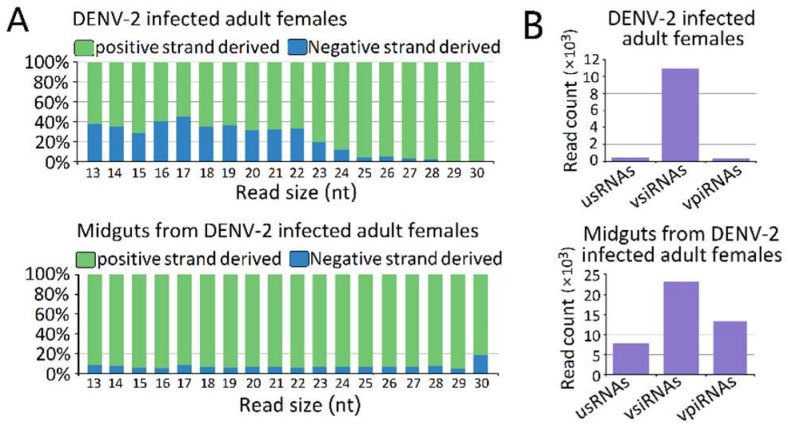
Characterization of virus-derived small RNAs (vsRNAs). (**A**) vsRNA strand preference of DENV-2-infected adult females and midguts from DENV-2-infected adult females; (**B**) Abundances of three classes of DENV-2 derived vsRNA from DENV-2-infected adult females and midguts from DENV-2-infected adult females. Mean vsRNA distribution by sRNA size group.

The vsRNAs in the DENV-2-infected adult females (Figure 3A) and the DENV-2-infected female midgut libraries (Figure 3B) showed obviously positive (genome) strand biases of 68.14% and 93.56% positive-sense vsRNAs, respectively. In the DENV-2-infected female midgut libraries, the ratio of plus- to minus-strand-derived vsRNAs was relatively stable across the 13–30 nt length. However, in DENV-2-infected adult females, the ratio of positive-sense-derived vsRNAs peaked at 17 nt in length and increased from 22 nt, following the increased length of vsRNAs. In particular, for vsRNAs from 25 to 29 nt in length in the DENV-2-infected adult females libraries, nearly 96.27% of the vsRNAs found were derived from the positive-sense (Figure 3A).

### 3.3. Genome Distribution of DENV-2-Derived sRNAs

**Figure 4:**
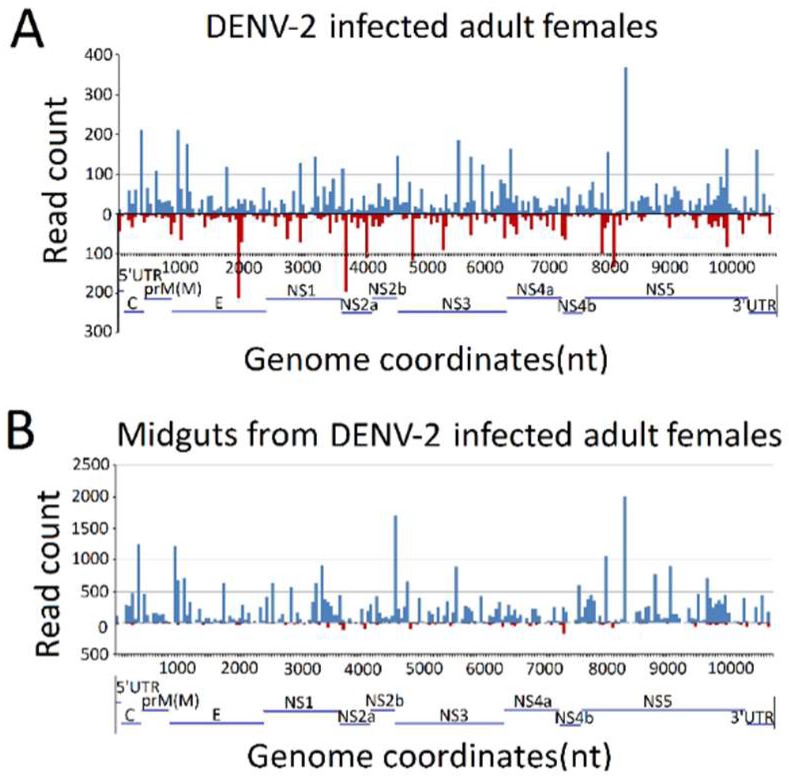
Frequency map of the distance between 13–30 nt virus-derived small RNAs (vsRNAs) that mapped to strands of the dengue virus 2 (DENV-2) genome. (**A**) DENV-2-infected adult females; (**B**) midguts from DENV-2-infected adult females. vsRNAs that mapped to the positive and negative strands of the DENV-2 genome are shown in blue and red, respectively. The organization of the DENV-2 genome is shown in the lower panel.

To explore the origins of vsRNAs, we focused on the vsRNAs’ genome locations by matching vsRNAs with the DENV-2 genome. The results indicated that vsRNAs in both DENV-2-infected adult females and DENV-2-infected female midgut libraries were not evenly distributed, but rather, were clustered in a few defined regions of the DENV-2 genome, especially in several hot spots. In DENV-2-infected adult females, several vsRNA hot spots originated from either negative-sense or positive-sense vsRNA, whereas, in the DENV-2-infected female midgut libraries, hot spots were mainly produced from the positive-strand virus genome, and only a few lower abundance vsRNAs accumulated in regions in the negative strand. It is notable that there was a high abundance of reads from one concentrated at a point around 8200 nt (Figure 4), located at nonstructural protein 5 (NS5) of the DENV-2 genome, in both libraries. To further detect whether these hot spot-derived vsRNAs had some relation to secondary structures of DENV-2 genomic RNA structures and the complementary strand, and whether these RNA secondary structures could act as dsRNA substrates for Dcr2, we analyzed potential secondary structures in the DENV-2 genomic RNA and its reverse complementary strand using RNAfold [57]; as a result, most of these predicted local double-stranded stem regions lacked precise base pairing over >21 bp. Hence, it was inappropriate to correlate these secondary structures with vsRNA hot spots.

### 3.4. Characterization of DENV-2-Derived vpiRNA

**Figure 5:**
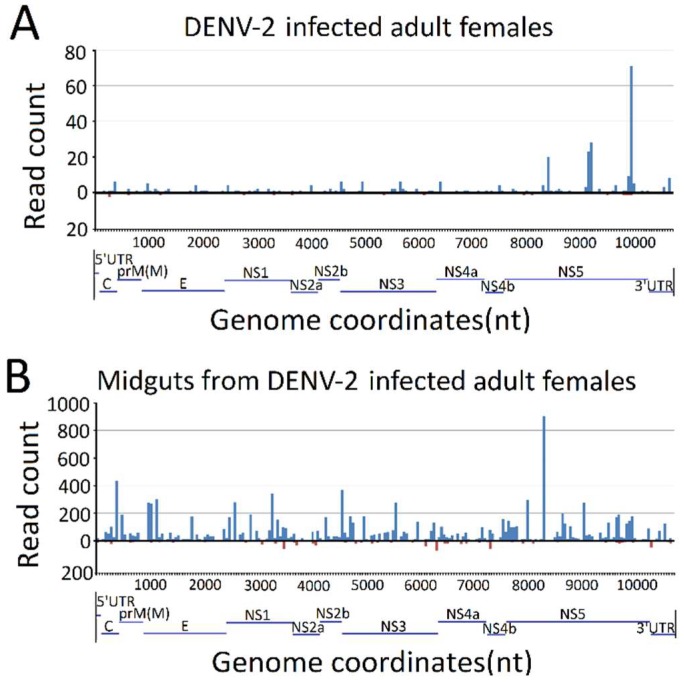
The virus derived piRNAs (vpiRNA) genome coverage distribution in Dengue virus 2 (DENV-2)-infected *Aedes albopictus* and midgut. (**A**) DENV-2-infected adult females; (**B**) midguts from DENV-2-infected adult females. vsRNAs that mapped to the positive and negative strand of the DENV-2genome are shown in blue and red, respectively. Organization of the DENV-2 genome is shown in the lower panel.

We next grouped these vsRNAs into three classes, based on their lengths. The first group was usRNAs (13–19 nt); the second group had a higher abundance of 20–23 nt vsiRNAs in both libraries, which depended on dicer for their biogenesis; and the third group was approximately 24–30 nt in length, as candidates of vpiRNAs (Figure 3B). We focused on the 24–30 nt vpiRNAs. Similar to vsRNAs, vpiRNAs also displayed uneven genome distributions and were enriched at several hot spots. In DENV-2-infected adult females, vpiRNAs peaked at the 3′ end of NS5 of the viral genomic RNA (Figure 5), whereas the peak was in the middle region of NS5 of the viral genomic RNA in DENV-2-infected female midguts. These predicted vpiRNAs exhibited a stronger strand bias, both in DENV-2-infected females and DENV-2-infected female midgut libraries, with more than 94.99% and 93.43% of the reads mapping to the viral plus-strand genomic RNA, respectively. The vpiRNAs detected in both libraries are listed in Table S5. We also analyzed the genomic distribution of usRNAs and vsiRNAs. Compared to vpiRNAs, vsiRNAs displayed a similar genomic distribution, and the major hot spots were located at the NS5 region in DENV-2-infected adult females, as well as the NS3 and NS5 regions in DENV-2-infected female midguts (Figure S1). However, usRNAs showed more irregular distribution patterns throughout the genome, especially in DENV-2-infected female midguts (Figure S1).

**Figure 6:**
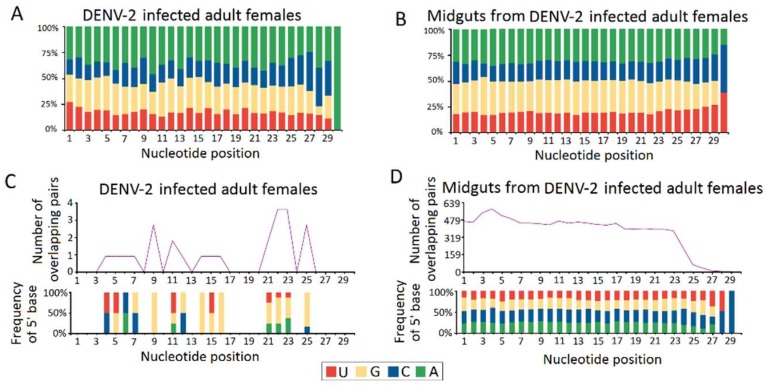
Characterization of virus derived P-element induced wimpy testis (PIWI)-interacting RNA (piRNAs) (vpiRNA). The upper panel (**A**,**B**) shows the base composition of repeat-derived piRNAs of DENV-2-infected adult females and DENV-2-infected female midguts. The X-axis represents the nucleotide positions relative to the 5′ ends of the piRNAs. The Y-axis represents the percentage of base bias. The lower panel (**C**,**D**) shows ping-pong pair analyses of DENV-2-infected adult females and DENV-2-infected female midguts. The length of overlap is shown on the horizontal axes. Indicated above each axis is the number of possible overlapping pairs of small RNAs with the specified overlap size. Indicated below each axis is the relative frequency of the 5′ base identity for overlapping sequences. The color code for the bases is indicated in the centre box.

Except for the vpiRNAs in the DENV-2-infected adult females libraries having a weak preference for adenine at position 10 (10A) (43.31%) as the feature of secondary sense piRNA (Figure 6A), these 10A sense piRNAs were thought to have originated from the ping-pong amplification cycle in an Aub slicer-dependent manner. However, a uridine at the first position (5U), as a key characteristic of primary antisense piRNAs was not observed in DENV-2-infected adult females (Figure 6A) and DENV-2-infected female midguts (Figure 6B). Furthermore, a significant 10 nt overlap (ping pong signal) between sense and antisense vpiRNA were also absent in DENV-2-infected adult females (Figure 6C) and DENV-2-infected female midguts (Figure 6D). These results indicated that vpiRNA biogenesis are involved in non-canonical pathways.

### 3.5. Characterization of vepiRNAs

To explore whether host piRNAs are generated in the process of host–virus interaction, we compared the uniquely-matched piRNA profiles of DENV-2-infected samples to uninfected controls, based on normalized expression levels (TPM).

In the DENV-2-infected adult female libraries, the vast majority of vepiRNAs were derived from intergenic regions (57.76%) with no known functions, then repetitive elements (35.93%), followed by gene regions (6.31%). About 65.92% of repeat piRNAs were derived from Class I TEs (retrotransposons), which included 34.97% and 30.95% of repeat piRNAs derived from long interspersed nuclear elements (LINEs) and long terminal repeats (LTRs), respectively, whereas 32.91% of piRNAs were mapped to class II TEs (DNA transposons). The typical strand bias was displayed in repetitive element-derived piRNAs; the majority of them were derived from the antisense strand. The sense/antisense ratios of DNA transposons, LINEs, and LTR-derived piRNAs were 0.02, 0.22, and 0.40, respectively. Furthermore, piRNAs in DNA transposons, LINEs, and LTRs showed strong 1U biases (89.39%, 97.58%, and 91.40%, respectively); however, they lacked a typical elevation for 10A (18.18%, 30.91%, and 32.62%, respectively).

A total of 61.44% of gene-derived piRNAs were matched to exon regions, and 38.56% were mapped to introns, which strongly suggests that gene-derived piRNAs are generated from precursor mRNA (pre-mRNA). The sense/antisense ratios of exon- and intron-derived piRNAs were 0.10 and 0.52, respectively. Exon- and intron-derived piRNAs also showed a strong 1U bias (82.42% and 94.37%, respectively), but no obvious elevations for 10A (28.57% and 21.12%, respectively).

Moreover, we introduced a threshold of 70 read counts, and these relatively highly-expressed piRNAs were analyzed further. Based on Poisson distribution statistics, a total of 1851 piRNAs were regarded as differential expressed vepiRNA candidates (*p* < 0.01, FDR < 0.01, and log2 fold change >1 or <−1) between DENV-2-infected adult females and uninfected female adults. A total of 22 piRNAs were regarded as differential expressed vepiRNA candidates between the DENV-2-infected female midguts and uninfected female midguts (Table S6). From the Poisson analysis of single sRNA library, selected vepiRNA were used for further qRT-PCR analysis using three biological replicates. 

### 3.6. Validation of piRNA Expression Using Stem-Loop qRT-PCR Analysis

**Figure 7:**
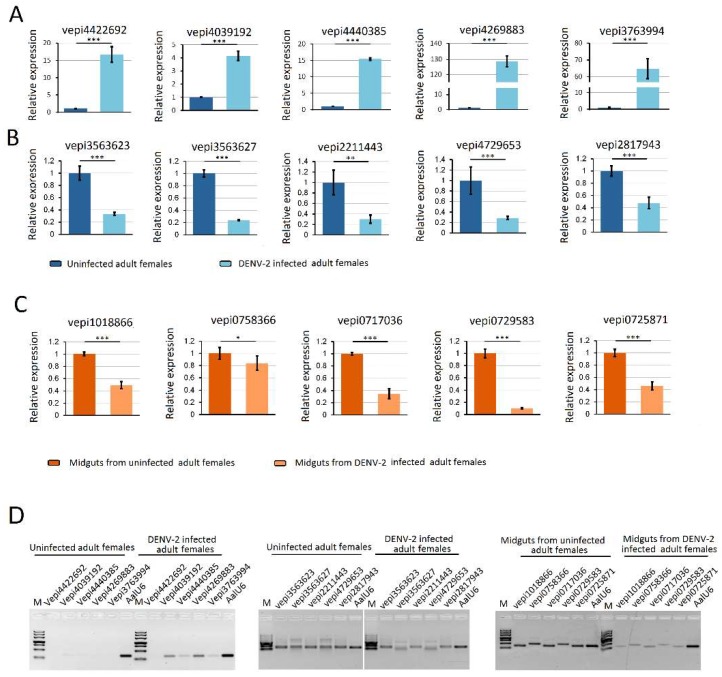
Quantification of DENV-2 infection induced host endogenous differentially expressed piRNAs (vepiRNAs) by stem–loop RT–PCR. (**A**) quantification of DENV-2 infection induced upregulated vepiRNA expression level in DENV-2 infected adult females; (**B**) quantification of DENV-2 infection induced downregulated vepiRNA expression level in DENV-2 infected adult females; (**C**) quantification of DENV-2 infection induced downregulated vepiRNA expression level in midguts from DENV-2 infected adult females; (**D**) The PCR products were run on 3% agarose gel in 1X TBE stained with ethidium bromide. *: *p* < 0.05; **: *p* < 0.01; ***: *p* < 0.001.

**Figure 8:**
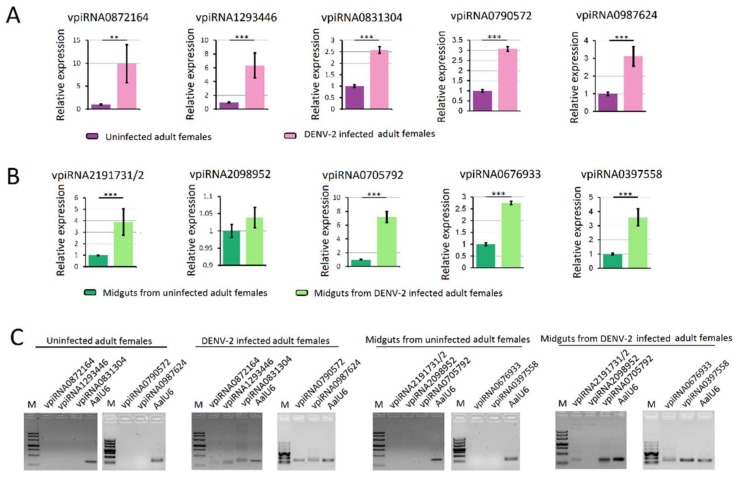
Quantification of DENV-2 derived piRNAs (vpiRNA) by stem-loop RT-PCR. (**A**) quantification of vpiRNA expression level in DENV-2 infected adult females; (**B**) quantification of vpiRNA expression level in midguts from DENV-2 infected adult females; (**C**) The PCR products were run on 3% agarose gel in 1X TBE stained with ethidium bromide. Note: vpiRNA2191731, vpiRNA2191732, vpiRNA0705792, vpiRNA0705793, vpiRNA3130226 and vpiRNA0705787 differed by only a few nucleotide base lengths at the 5′ and 3′ ends. A similar pattern was also found in vpiRNA0676933 and vpiRNA1747127. **: *p* < 0.01; ***: *p* < 0.001.

RNA-seq results from DENV-2 infected adult females were validated by using vepiRNAs based on fold-change analyses, followed by gene quantitative analysis using stem-loop qRT-PCR (top 5 in Table S6-1: Vepi4422692, Vepi4039192, Vepi4440385, Vepi4269883, and Vepi3763994; top 5 in Table S6-2: vepi3563623, vepi3563627, vepi2211443, vepi4729653 and vepi2817943; top 5 in Table S6-3: vepi1018866, vepi0758366, vepi0717036, vepi0729583 and vepi0725871). Moreover, the top five vipRNAs expressed in the DENV-2-infected adult females (Table S5-2: vpiRNA0872164, vpiRNA1293446, vpiRNA0831304, vpiRNA0790572 and vpiRNA0987624) and DENV-2-infected female midguts (Table S5-1: vpiRNA2191731/vpiRNA2191732, vpiRNA2098952, vpiRNA0705792, vpiRNA0676933 and vpiRNA0397558) (Table S5) were also selected to validate the expression level by stem-loop qRT-PCR method (described above). As a result, all of the candidate vepiRNAs (Figure 7A–C) and vpiRNAs (Figure 8A,B) were expressed at significantly higher levels in DENV-2 infected libraries, except vpiRNA2098952. This result was further confirmed by qPCR product gel electrophoresis and sequencing, as shown in Figure 7D and Figure 8C; all of the selected vepiRNAs and vpiRNAs were shown to have distinct specific expression patterns induced by DENV-2 infection, but vepi4422692 and vpiRNA2098952 were invisible on an ethidium bromide (EtBr) stained gel.

### 3.7. PIWI Protein Family Members of Adult Females and Their Expression Profiles

A tBLASTx search of the *Ae. albopictus* genome and transcripts, using the *Ae. aegyti* PIWI protein family genes as queries, was performed. Significant gene expansion was observed in PIWI sub-family proteins of *Ae. albopictus* compared to *D. melanogaster*, with an e-value of less than 1 × 10^−10^: one Armitage ortholog (AALF004386); one Spindle-E ortholog (AALF006846); 12 Rm62-like orthologs (AALF027261, AALF027794, AALF018740, AALF019569, AALF020841, AALF025200, AALF007978, AALF007782, AALF013981, AALF016979, AALF022327, AALF025597); two Ago3 orthologs (AALF025916, AALF025919); six Ago4-like orthologs(AALF006534, AALF006708, AALF005498, AALF005499, AALF006337, AALF008582); and three Ago4-like orthologs (AALF016369, AALF007445, AALF015479) (Table S7).

**Figure 9:**
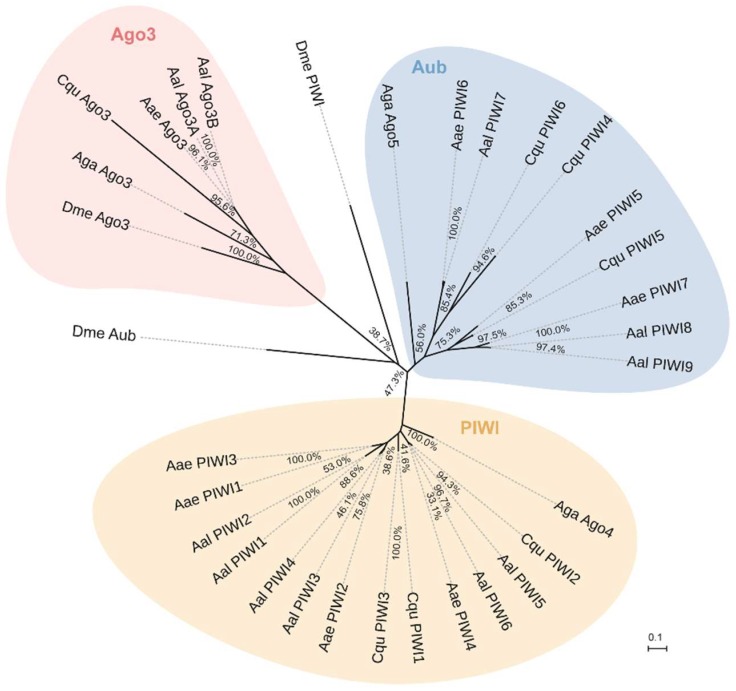
Phylogenetic and molecular evolutionary analyses of PIWI protein family members of mosquitoes. Bootstrap values for 1000 replicate analyses are shown at the branching points. The bar at the bottom indicates the branch length, corresponding to the mean number of differences (0.05) per residue along each branch. Gene ID refers to Table S7.

The topology obtained for the PIWI protein family protein phylogeny is shown in Figure 9. Phylogenetic analyses showed high confidence levels in the defined groups. As expected, based on phylogenetic relationships, three well-supported clades were obviously displayed, as described previously [45,58]. Ago3s of three genera of mosquitoes formed a single clade with *D. melanogaster. Ae. albopictus* PIWI1–6 were grouped into a clade with *An. gambiae* Ago4, whereas the PIWI7–9 were grouped into a clade with *An. gambiae* Ago5 (Figure 9).

**Figure 10:**
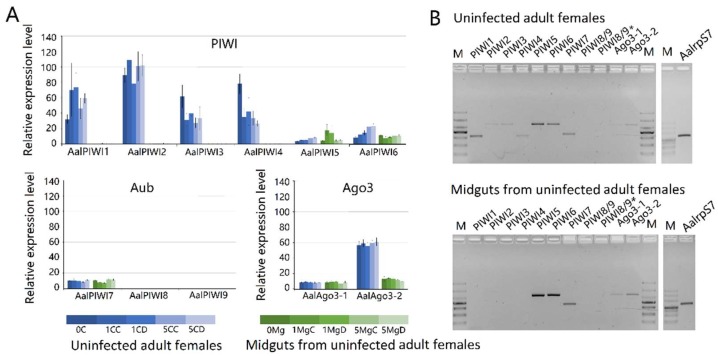
Expression profiles of PIWI protein family members of *Aedes albopictus*. (**A**) RNA-seq expression profile. tBLASTx was performed against *Ae. albopictus* and midgut RNA-seq data spanning serial time points post-DENV-2 infection (SRP077936). Counts were normalized to the number of million reads per sample; (**B**) RT-PCR spatial expression analysis of PIWI protein family member genes. *Ae. albopictus* Rps7 was used as an internal control. The analyses were performed on the following samples: 0C: uninfected carcass; 0Mg: uninfected midgut; 1MgC: control midgut at 1 dpi; 1MgD: DENV-infected midgut at 1 dpi; 1CC: control carcass at 1 dpi; 1CD: DENV-infected carcass at 1 dpi; 5MgC: control midgut at 5 dpi; 5MgD: DENV-infected midgut at 5 dpi; 5CC: control carcass at 5 dpi; 5CD: DENV-infected carcass at 5 dpi.

Results based on RNA-seq (Figure 10A) and RT-PCR (Figure 10B) analyses indicated that transcripts of PIWI1–7 and Ago3(1–2) are readily detected in *Ae. albopitus* adult females, whereas Piwi8/9 is considerably lower. Different from the carcass, in the midgut, only PIWI5–7 and Ago3(1–2) were visibly detected (Figure 9). Interestingly, PIWI8 and 9 transcripts were not detected in both the midgut and mosquito whole-body using RT-PCR and RNA-Seq analyses, so we continued to explore the temporal expression patterns of PIWI genes further using the RT-PCR method. As a result, Piwi8 and 9 were highly expressed in the embryo stage, whereas Piwi1–4 were shown to have a higher expression level in embryos, adult males, and in post blood meal (PBM) females (Figure S2). Moreover, DENV-2 infection showed no obvious effects on the transcription levels of PIWI5–7 and Ago3.

## 4. Discussion

Our current understanding of the primary role of the piRNA pathway fulfils a vital function in the preservation of genomic integrity in animal germ cells against invasive transposable elements (TEs) [59]. Furthermore, some non-repetitive and non-transposon-related piRNAs may perform crucial functions in post-transcriptional regulation. For example, piRNA is considered to regulate the transcription of non-repetitive, protein-coding genes in *D. melanogaster*, and is involved in the sex determination hierarchy in *Bombyx mori*.

Recently, sRNA deep sequencing revealed a class of viral sequence-derived piRNAs from several infected insects and in insect cell lines [1,3,4,5,6,7,15,16,17,18,19,20]. vpiRNA of insects was first reported using high-throughput sequencing data of *Drosophila* somatic OSS cells, persistently infected with six RNA viruses, including double-strand RNA (dsRNA) viruses: Drosophila X virus (DXV), American nodavirus (ANV), Drosophila birnavirus (DBV), and Drosophila tetravirus (DTrV), as well as positive-sense single-stranded RNA (+ssRNA) viruses: Drosophila C virus (DCV) and Norovirus (NV) [47]. However, in adult flies, vpiRNAs have not been found [60]. For mosquitoes, vpiRNAs have not been found in the *Anopheles* and *Culex* genera, post-infection with several alphaviruses and flaviviruses, including WNV [37], O’nyong-nyong virus (ONNV) [61], or Usutu virus (USUV) [62]. However, vpiRNAs are regularly discovered in several arboviruses (including members of the *Togaviridae* [21,22,23,24,25,26], *Flaviviridae* [27,28,29], *Bunyaviridae* [21,22,30,31,32], and *Reoviridae* [27] families) infected mosquitoes and cell lines. Furthermore, newly-published research suggests that the piRNA pathway does not seem to be involved in flies’ antiviral defense, as PIWI proteins are largely confined to *Drosophila* gonads [63,64,65,66], and the lack of the necessary piRNA pathway components in somatic cells, which increases *Drosophila’s* susceptibility to viral infections. In contrast, the expanded family of PIWI proteins is expressed in somatic tissues in *Ae. Aegypti* [67], and the functional links among the piRNA pathway and arbovirus replication [24,25] have also been described. These results further strengthen the evidence that the piRNA pathway contributes to antiviral defense in *Aedes* mosquitos.

*Ae. albopictus* is ranked at number four on the list of 100 worst invasive species on Earth [57]. *Ae. albopictus* exhibits excellent adaptability to a broad spectrum of environmental conditions, is phenotypically polymorphic, and displays variations in its vectorial capacity for mosquito-borne viruses (MBV) [34,35,36]; it has a very large genome (1967 megabase pairs, Mbp) and is 1.43 times larger than the *Ae. aegypti* genome (1376 Mbp) [37]. Only virus-derived vsRNA profiles of infected *Ae. albopictus* C6/36 cells have been previously reported [21,22,23], but both adult female whole bodies and their midguts, which act as the first barrier for pathogen transmission, still lack supporting data.

The extrinsic incubation period (EIP) corresponds to the time necessary for a virus to reach mosquito saliva after an infectious blood meal [63]. For DENV, viral particles started to be detected in the salivary glands of *Aedes* mosquitos at approximately 9 dpi [68]. On the other hand, DENV had not completely passed through the midgut barrier at 1 dpi. Furthermore, vpiRNAs were visible at 1 dpi in SINV-infected Aag2 cells, when infection was fully established. Thus, we analyzed the sRNA transcriptome at these time points.

Three pathways were thought to be involved in the antivirus immune responses in mosquito midguts. First, the cytokine-activated Janus kinase (JAK)—signal transducer and activator of the transcription (STAT) signalling pathway in mosquitoes—was primarily observed in the *Ae. aegypti* midgut [69]. Transient impairment of the JAK/STAT pathway via RNAi-mediated gene silencing led to increased DENV titres in *Ae. aegypti* midguts. Second, the exogenous siRNA pathway showed no evidence of involvement in antiviral responses in the midgut of ONNV-infected *An. Gambiae* [70], and downregulation of SRRP key factors, Ago2, or Dicer2, promoted viral duplication in the midguts of flavivirus- or alphavirus-infected *Ae. Aegypti* [71,72,73]. Third, certain miRNAs were also confirmed to definitely influence DENV replication in *Aedes* mosquito midguts [38,39]. The abovementioned results indicate that the exogenous (exo-) siRNA pathway, JAK-STAT pathway, and miRNA pathway play important roles in the regulation of arbovirus replication in the midgut.

In our work, three types of vsRNA, usRNAs, vsiRNAs, and vpiRNA were detected in both DENV-2-infected female *Ae. albopictus* and midgut libraries. As expected, the exogenous vsiRNAs were more abundant in vsRNA, suggesting that vsiRNA-mediated RNA still played an important role in anti-DENV-2 defense function, both for the whole body and midgut of *Ae. albopictus.* In particular, vpiRNA was second in abundance, next to vsiRNA, in virus-infected midguts. However, the role of these DENV-2-specific vpiRNAs in the anti-DENV-2 response still need further investigation.

In most +ssRNA mosquito-borne viruses (alphaviruses and flaviviruses), the plus viral genomic RNA strand that corresponds to the viral transcript is over-represented compared to the minus strand [18,19,20,21,22,23]. Similar results were obtained in the present study; both vsRNA and vpiRNA showed obvious strand bias, and were mainly produced from the positive strand of the DENV-2 genome, which was in accordance with the relative amounts of plus and minus strands of DENV-2. vpiRNA production was mostly confined to specific hot spot regions in the DENV-2 genome, especially in the NS5 gene region, which showed similar results to piRNA hot spots in DENV-2 and cell-fusing agent virus (CFAV)-infected *Ae. aeagyti* and Aag2 cells [24]. In alphaviruses, such as SINV, Chikungunya virus (CHIKV), and Semliki Forest virus (SFV), vpiRNAs also displayed extreme strand bias towards sequences from the viral plus strands, but these alphavirus-derived vpiRNAs were predominantly produced from a subgenomic RNA that was transcribed from an internal promoter sequence [20]. The origins of these piRNA spikes remain unclear, but this difference strongly indicates that the mechanisms underlying alphavirus and flavivirus vpiRNA biogenesis are essentially variant.

The propensity for a “5U”, as a distinct characteristic of primary piRNAs, was not observed in vpiRNA for both the DENV-2-infected adult females and the DENV-2-infected female midgut libraries. However, in the DENV-2-infected adult female libraries, vpiRNA showed a weak preference for 10A as a feature of secondary piRNA. Moreover, these vpiRNAs did not exhibit a typical ping-pong signature. These results were similar to those previously described in DENV and cell fusing agent virus (CFAV)-infected mosquito cell lines and *Ae. Aegyti* [6,24,25,26]. However, the major characteristics of 5U and 10A bias as well as ping-pong signature, were observed in SINV-, CHIKV-, and SFV-infected mosquitoes and cell lines [18,19,20,21,22,23]. Thus, it is noted that these flaviviruses vpiRNAs may use a unique biogenesis pathway that is distinct from that of alphaviruses. Although flavivirus vpiRNAs exhibit the secondary piRNA features, whether a different, amplification-independent mechanism is responsible for flavivirus vpiRNA production remains to be determined.

As previously described [22], the vpiRNA-associated proteins, Piwi5 and Ago3, were predominantly expressed in the cytoplasm of Aag2 cells. Thus, as +ssRNA virus RNA replication occurs in the cytoplasm, and generally does not enter the nucleus, the key biogenesis factors of vpiRNA and viral RNAs are co-expressed in the cytoplasm. In fact, the vast majority of vpiRNAs were also present in the cytoplasmic fraction, indicating that vpiRNA biogenesis occurs in the cytoplasm of mosquito cells. Thus, it is supposed that vpiRNA production is a purely cytoplasmic event, and other unknown mechanisms must be involved in vpiRNA biogenesis.

It has also been previously shown that vpiRNA-like small RNAs are capable of modulating the pathogenesis of alphavirus infections in dicer-2 null mutant mosquito cell lines, and are defective in viral siRNA production [20]. In addition to vpiRNA, host endogenous piRNAs were thought to also be involved in the anti-viral response. For example, the knockdown of *Piwi4* (a member of the *PIWI* gene family) does not cause a reduction in vpiRNA levels [21], but, rather, enhances the replication of SFV in Aag2 cells [21].

We identified 1479 vepiRNAs displaying an up-regulated expression in the DENV-2-infected adult female library; however, no up-regulated expression piRNAs were found in DENV-2-infected female midguts. Thus, we analyzed the PIWI protein family members of adult females and their expression profiles. In the midgut, only Piwi5–7 and Ago3(1–2) were readily detected in every midgut sample. As previously reports note, the biogenesis of TE-derived host piRNA, either direct or indirect, requires nearly all PIWI protein activity, which suggests the lack of necessary PIWI proteins as a reason for vepiRNA deficiency in the midgut [22]. Furthermore, the 10A bias, as a feature of secondary piRNA, does not appear in midgut vpiRNAs, which indicates that the lack of certain PIWI proteins could lead to the loss of the vpiRNA biogenesis pathway in the midgut, compared to other tissues. Whether these 24–31 nt DENV-2-derived small RNA originated from the PIWI process or not still needs to be further explored. As previously described, Piwi1 and Piwi3 were highly expressed specifically in the ovaries of mosquitoes, a tissue that was generally not infected by SINV [74,75]. The ovaries were also less susceptible to DENV-2 infection than other tissues [76], Piwi1–3 may be associated with viral dissemination in mosquitoes. The role of Piwi1-3 in antivirus immunity requires further investigation.

Except for intergenic regions, major vepiRNAs are derived from Class I TEs (retrotransposons); moreover, the majority of them are derived from the antisense strand. In addition to retroviruses, RNA-only viruses have also given rise to transposons in many mosquito genomes, although the specific mechanisms involved in this horizontal RNA-to-DNA information flow are less clear. The exogenous non-self nucleic acids from infecting viruses can be targeted by piRNA-like RNAs, but this requires genetic information flow in an unexpected retrotransposon-dependent manner [77]. Thus, we deduced that vepiRNAs derived from TEs are antisense relative to the proposed ancient viral RNA, originally a response to interactions between infectious viruses and transposons.

## Supplementary Materials

The following are available online at http://www.mdpi.com/1999-4915/10/4/213/s1, Figure S1: The usRNA and vsiRNA genome coverage distribution in DENV-2-infected *Aedes albopictus* and midgut, Figure S2: RT-PCR developmental expression analysis of PIWI protein family members’ genes, Table S1: PCR programs and primers for RT-PCR, Table S2: PCR programs and primers for stem loop RT-PCR, Table S3: Small RNA sequence statistics for DENV2 infected and uninfected adult female and midgut libraries, Table S4: vsRNAs from DENV2-infected adult female and midgut libraries, Table S5: DENV-2 derived piRNA (vpiRNA) profiles in infected adult female and midgut libraries, Table S6: DENV-2 infection derived host piRNA (vepiRNA) profiles in infected adult female and midgut libraries, Table S7: PIWI protein family members in vector mosquitoes compared to drosophilids.
