# Supplementary figures and images for: piRNA Profiling of Dengue Virus Type 2-Infected Asian Tiger Mosquito and Midgut Tissues
Source: Viruses. 2018 Apr 22;10(4):213. doi: 10.3390/v10040213 (PMC5923507; doi:10.3390/v10040213)

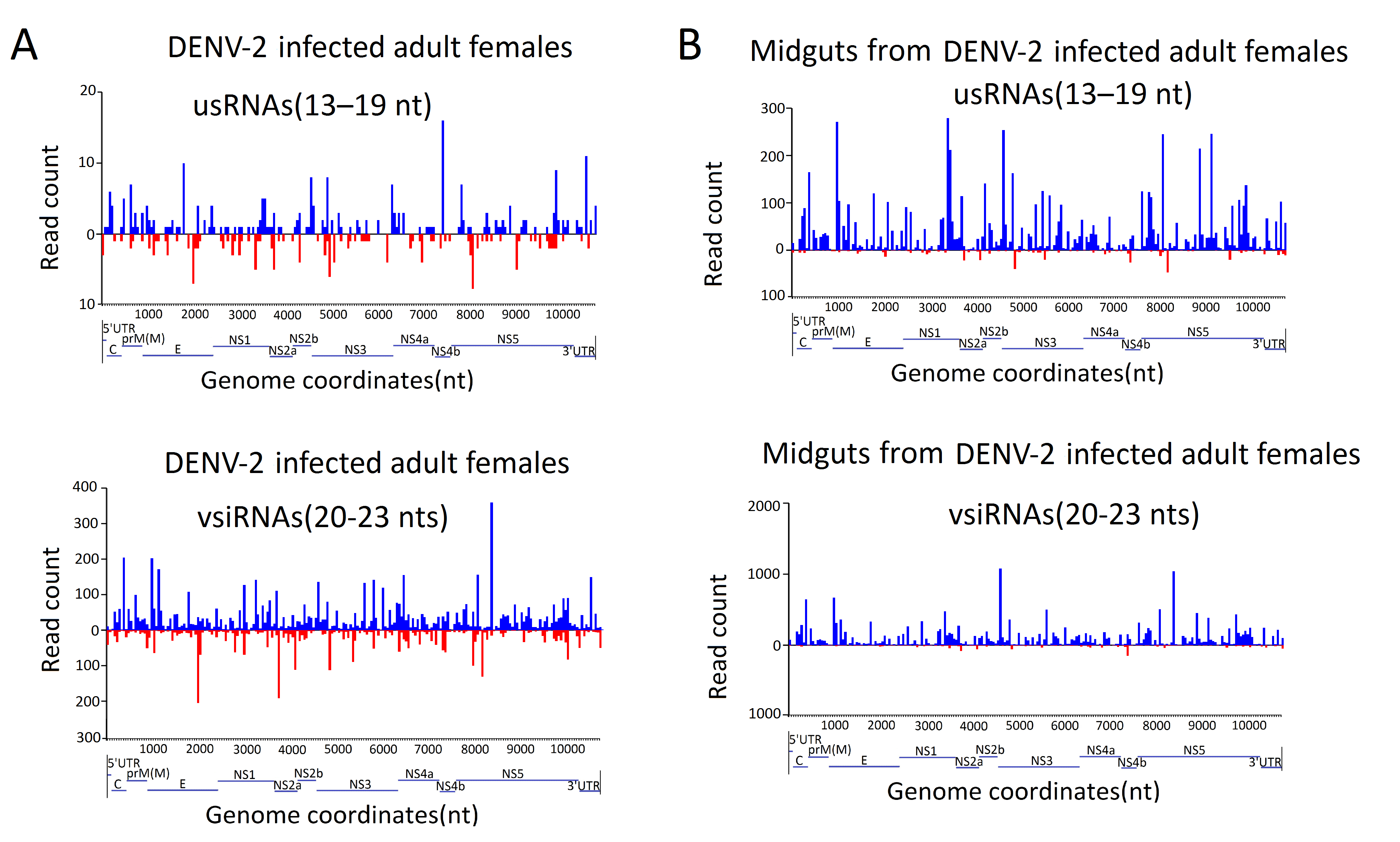

Supplement: Supplementary file 1 [file viruses-10-00213-s001.zip › viruses-276697 revise supplementary/Figure S1.tif]

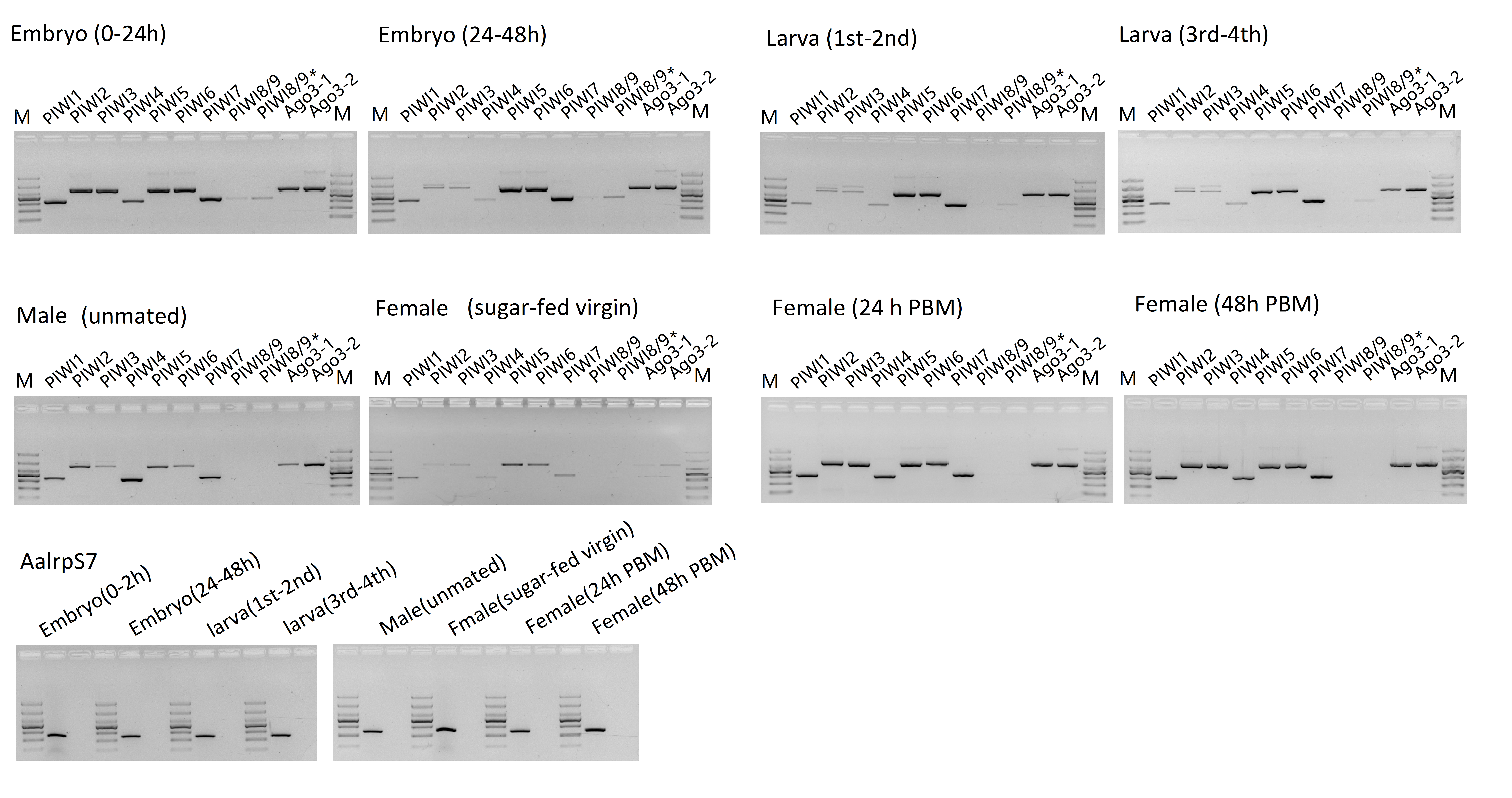

Supplement: Supplementary file 1 [file viruses-10-00213-s001.zip › viruses-276697 revise supplementary/Figure S2.tif]
